# Supplementary figures and images for: Short k-mer abundance profiles yield robust machine learning features and accurate classifiers for RNA viruses
Source: PLoS One. 2020 Sep 18;15(9):e0239381. doi: 10.1371/journal.pone.0239381 (PMC7500682; doi:10.1371/journal.pone.0239381)

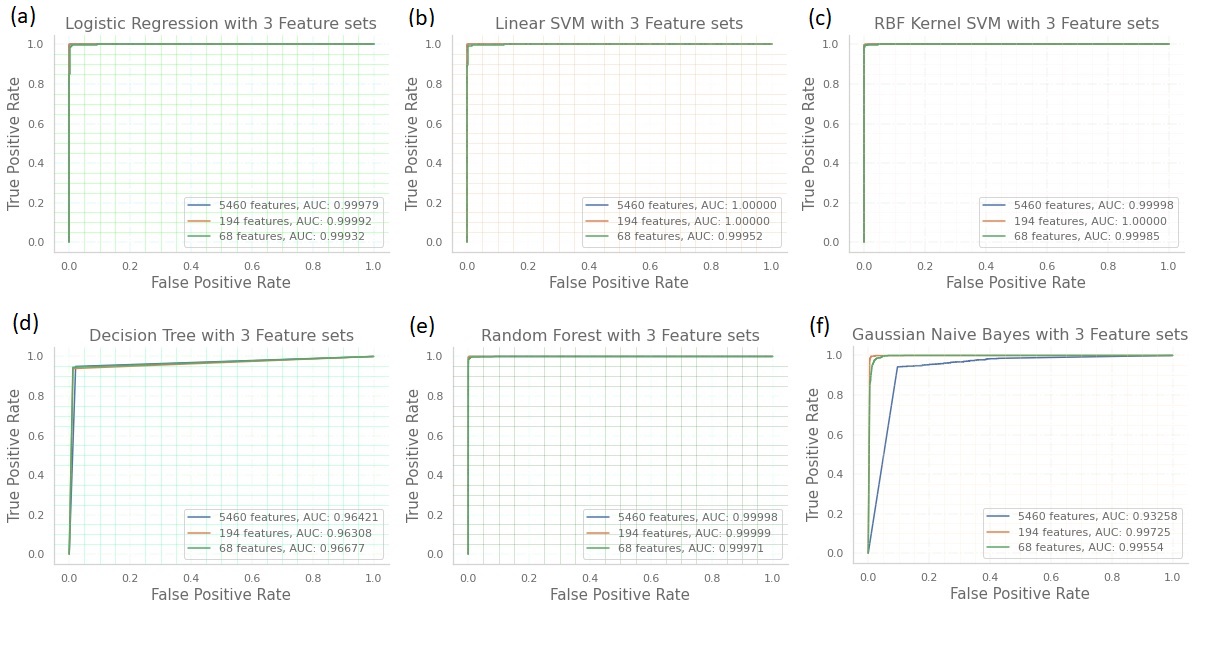

Supplement: S1 Fig — (a) Logistic Regression Classifier on 3 Feature Sets. (b) Linear SVM Classifier on 3 Feature Sets. (c) RBF-Kernel SVM Classifier on 3 Feature Sets. (d) Decision Tree Classifier on 3 Feature Sets. (e) Random Forest Classifier on 3 Feature Sets. (f) Gaussian Naïve Bayes Classifier on 3 Feature Sets. (JPG) [file pone.0239381.s001.jpg]
